# Comment on ‘Orthoplastic approach in the treatment of Gustilo–Anderson Type IIIB/C open tibial fractures: a consecutive 10-year series from China level I trauma center’

**DOI:** 10.1097/JS9.0000000000003189

**Published:** 2025-08-23

**Authors:** Kai Qiu, Hui Wang, Hong Zou

**Affiliations:** Department of Knee Joint, Mianyang Orthopedic Hospital, Sichuan, China

*Dear Editor,*

We read with great interest the recent publication entitled “Orthoplastic management of Gustilo–Anderson Type IIIB/C open tibial fractures: a consecutive 10-year series from China level I trauma center” published in your esteemed journal[1]. The findings of this study, especially the dramatic decrease in infection rates, nonunion rate, and better functional results, highlight the paradigm shift this combined approach constitutes in limb salvage. Despite the valuable insights the authors present, there are some crucial aspects that require clarification. We have ensured that this correspondence complies with the TITAN Guidelines 2025-governing declaration and use of AI[2].

First, it is interesting to note the lack of a clear statistically significant difference in fracture healing time between the orthoplastic and orthopedic groups[1]. While the orthoplastic approach evidently reduces complications that delay healing, this specific measure suggests that for high-energy Gustilo–Anderson Type IIIB/C fractures, there is an inherent biological timeline that cannot be decreased significantly even with ideal surgical methods. This result calls for the critical re-evaluation of time to union as the sole primary measure of outcome. Future research must turn its attention to assessing the quality of bone healing, rather than merely its speed. This requires the application of advanced imaging modalities and the exploration of bone biological markers^[3,4]^.

Second, the results of the study show that plate fixation, when employed in the orthoplastic context, yields infection rates comparable with those of intramedullary nailing, thus challenging current dogma[1]. This suggests that the meticulous, single-stage soft tissue coverage inherent in the orthoplastic approach successfully overcomes the traditional infection risks traditionally associated with plates in the setting of contaminated open fractures. If this paradigm shift is consistently confirmed, it would allow for increased surgical flexibility[5]. Both to confirm this finding and to investigate long-term mechanical stability, implant survival, and re-operation rates for the two fixation methods in this specific setting, multi-center, prospective trials are necessary.

Finally, as the study strengthens the effectiveness of local antibiotics in minimizing infection rates[1], the controversies surrounding it still call for further debate[6]. Issues of the possibility of encouraging antibiotic resistance, especially the development of resistant strains, continue to be a concern.

## Data Availability

All data are included in the article.
